# Classical and Non-Classical Progesterone Signaling in Breast Cancers

**DOI:** 10.3390/cancers12092440

**Published:** 2020-08-27

**Authors:** Diego A. Pedroza, Ramadevi Subramani, Rajkumar Lakshmanaswamy

**Affiliations:** 1 Graduate School of Biomedical Sciences, Texas Tech University Health Sciences Center El Paso, El Paso, TX 79905, USA; diego.a.pedroza@ttuhsc.edu (D.A.P.); ramadevi.subramani@ttuhsc.edu (R.S.); 2 Center of Emphasis in Cancer, Department of Molecular and Translational Medicine, Paul L. Foster School of Medicine, Texas Tech University Health Sciences Center El Paso, El Paso, TX 79905, USA

**Keywords:** progesterone, classical signaling, non-classical signaling, breast cancer, progesterone receptor, membrane associated progesterone receptors, membrane progesterone receptors, progesterone receptor membrane component 1

## Abstract

Much emphasis is placed on estrogen (E2) and estrogen receptor (ER) signaling as most research is focused on understanding E2 and ER’s ability to enhance proliferative signals in breast cancers. Progesterone (P4) is important for normal mammary gland development, function and menstrual control. However, P4 and its receptors (PRs) in breast cancer etiology continue to be understudied and its role in breast cancer remains controversial. The Women’s Health Initiative (WHI) clinical trial clearly demonstrated the importance of progestogens in breast cancer development. P4 has historically been associated with classical-signaling through nuclear receptors, however non-classical P4 signaling via membrane receptors has been described. Progestogens have the ability to bind to nuclear and membrane receptors and studies have demonstrated that both can promote breast cancer cell proliferation and breast tumor growth. In this review, we attempt to understand the classical and non-classical signaling role of P4 in breast cancers because both nuclear and membrane receptors could become viable therapeutic options for breast cancer patients.

## 1. Introduction

Breast cancer is the most frequently diagnosed cancer in women worldwide. It is estimated that approximately 2.1 million women will be diagnosed with breast cancer and over 626,000 women will die due to breast cancer globally per year [1]. In the USA 276,480 women, are expected to be diagnosed with breast cancer with an associated mortality of 42,170 in 2020 [2]. In perspective, one in eight USA women will be diagno, there are also multiple factors that are thought to be protective against breast cancers such as, early pregnancy, estrogen-only hormone sed with invasive breast cancer in her lifetime [2]. Risk factors for breast cancer include family history, BRCA1 and BRCA2 gene mutations, radiation exposure, body mass index, early menarche and or late menopause as well as long-term usage of combined hormone replacement therapy (HRT) [3,4,5,6,7,8]. Howevertherapy in hysterectomized women and risk-reducing mastectomy [9,10,11]. Breast cancers are clinically identified by the histopathological presence or absence of the estrogen receptor (ER), progesterone receptor (PR) and human epidermal growth factor receptor 2 (HER2) [12]. Upon diagnosis, breast cancer is classified into the following molecular intrinsic subtypes, Luminal A (ER+/PR+, HER2−), Luminal B (higher grade, ER+/PR+, HER2+/−) HER2-enriched and Basal-like (triple negative) [12]. Among them, luminal breast cancers are the least aggressive while HER2-enriched and Basal-like subtypes tend to have worse prognosis and lower overall survival outcomes [13,14]. Among these subtypes, luminal breast cancers account for over two thirds of all diagnosed breast cancers [15,16]. Treatment options for these breast cancers have mainly targeted ER and aromatase enzyme using the drugs Tamoxifen, Fulvestrant and Anastrozol [17,18]. However, clinical and epidemiological data demonstrate that a high percentage of women exhibit intrinsic resistance, and nearly all patients diagnosed with advanced disease and a significant amount with localized disease develop acquired or de novo resistance after initially responding to endocrine therapy [19,20,21].

A different subset of breast cancers that are characterized as ER+/PR− have been observed in patient tumor tissue and have been deemed as aggressive and tamoxifen resistant [22]. Lack of PR expression in breast cancer tumor tissues has been shown to be independently linked with worse prognosis [23]. Furthermore, these breast tumors have been associated with overall worse long-term outcome following neoadjuvant therapy [24]. A higher proliferation rate from increased S-phase fraction could be a reason as to why these tumors would be classified as luminal B like breast cancers [22,25]. Genetically these tumors have been shown to be highly unstable and possess increased DNA copy number gains compared to ER+/PR+ tumors [26]. It has even been suggested that ER+/PR− tumors have similar outcomes to that of Triple negative breast cancers (TNBCs) [27]. Furthermore, ER+/PR− tumors show higher nuclear grade, higher ki-67 levels, higher HER2 and Epidermal growth factor receptor (EGFR) expression compared to ER+/PR+ tumors [28].

Progesterone (P4) and PR have not been studied as extensively as estradiol (E2) and ER in treating breast cancers. The Women’s Health Initiative (WHI) study demonstrated that postmenopausal women who were treated with combined HRT consisting of conjugated equine estrogens plus the progestin Medroxyprogesterone Acetate (MPA) had an increased risk of breast cancer [17,29]. Overall a 26% increase of invasive breast cancer was observed in these women who were taking combined HRT [30]. In another arm of the WHI study, hysterectomized postmenopausal women were given estrogen alone replacement therapy. In this group, there was a 24% reduction in breast cancer incidence compared to hysterectomized postmenopausal women who received a placebo [31]. These findings demonstrate the significance of progesterone in breast cancer and it is important to understand its role to design and develop novel treatment strategies. In this review, we will explore the various aspects of progesterone signaling in breast cancer in the light of available literature.

## 2. P4 in Normal Breast Development

The mammary gland is a unique organ which is not fully developed at birth but actually begins to expand its rudimentary ductal system at puberty to its complete development after a women’s first full-term pregnancy [32]. In females, during puberty, the complex ductal system begins to develop in response to E2 and P4. The primary ductal epithelium begins to invade the mammary fat pad and gains further complexity during adulthood as ovarian hormones fluctuate throughout menstrual cycles [33,34]. The role of hormones in mammary gland development has been mainly established utilizing mouse models. Mouse knockout studies involving both ER and PR have demonstrated their implication in the development of the mammary gland [35]. In adult female ERα knockout (ERKO) mice, ductal elongation failed to form and did not respond to ovarian hormones [36]. In post-pubertal PR knockout mice (PRKO) ductal structures and ductal elongation was similar to that of wild type mice, but had reduced side branching [37]. Furthermore, when combined treatment of E2 and P4 was administered to PRKO and wild type mice, PRKO mice failed to respond, whereas the wild type mice responded with side branching and lobular development [38]. In peri-pubertal mice, P4 is able to promote the formation of tertiary side-branches on existing ductal networks [39]. In BALB/c mice, it has been shown that P4-dependent branching morphogenesis occurs in two phases. In the first phase, PR-dependent side branching relies on the activation of target genes involved in Rac-GTPase signaling and cyclin D1 [40,41]. In the second phase, lateral side branching associates with up-regulation of known P4 mediator receptor activator of nuclear factor kB ligand (RANKL) [41]. These data indicate that P4 signaling is complex and needs to be studied in depth to understand its role in breast cancer.

## 3. P4 Classical Signaling

**Figure 1:**
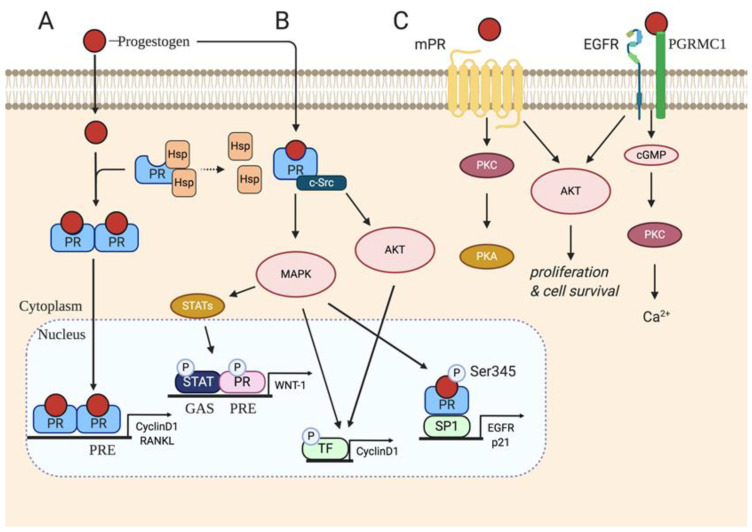
Overview of progesterone actions via classical and non-classical signaling. (**A**) Progestogen induced classical signaling to PRs leads to heat shock protein (HSP) dissociation, PR dimerization, progesterone response element (PRE)-binding and transcription of downstream effector targets, Cyclin D1 and RANKL. (**B**) Rapid progestogen non-classical activation of membrane-proximal actions on proto-oncogene tyrosine-protein Src (c-Src), mitogen activated protein kinase (MAPK) and protein kinase B (AKT) promotes PR phosphorylation and tethering to transcription factors that can then transcribe WNT-1, Cyclin D1, EGFR and p21. (**C**) Progestogens initiate non-classical signaling of membrane progesterone receptors (mPRs) and progesterone receptor membrane component 1 (PGRMC1) by activating downstream targets, protein kinase c (PKC), protein kinase a (PKA), cyclic guanosine monophosphate (cGMP) and AKT leading to Ca^2+^ influx, proliferation and cell survival. Figure 1 was created using Biorender.

Classical PRs are recognized as members of the nuclear receptor super-family of transcription factors, consisting of a DNA binding domain and a carboxyl-terminal ligand-binding domain [42]. PR-A and PR-B are the two main isoforms that are encoded from the same gene located on chromosome 11 (11q22-q23), and their transcription is controlled by distinct E2-induced promoters with alternative AUG initiation codons; hence PRs are thought to be direct targets of ERs [43]. The transcribed cytoplasmic PRs remain inactive and bound to a multi protein chaperone complex. Upon binding to P4 the PR can trigger multiple conformational changes including dimerization. The selective modifications induce dimerization of the two-ligand receptor protein complexes, which localize into the nucleus and bind to hormone response elements (HREs) more specifically to progesterone response elements (PREs). Interaction between the receptor complex consisting of transcription factors and co-activators, leads to the formation of a functional transcription initiation complex upon binding to specific target gene promoters [42] (Figure 1A).

In humans, multiple distinct progesterone receptor isoforms exist: PR-B, PR-A, PR-C, PR-M, PR-S and PR-T [44,45]. The truncated PR-A isoform lacks 164 amino acids from the amino-terminal domain, relative to the full receptor form PR-B and the N-terminally truncated PR-C isoform is translated beginning at residue 595 [46,47]. Novel truncated PR-M, lacking a DNA binding domain, has been described in the mitochondria while the existence of PR-S and PR-T remains controversial [45,48]. PR isoform signaling is tissue-selective, in PR-A KO mice, ablation of PR-A results in ovarian and uterine abnormalities with no effect to the mammary gland [49]. While in PR-B KO mice, PR-B ablation results in impaired mammary ductal morphogenesis with no affects to the ovaries, or uterus [49]. This suggest that in normal mammary gland function PR-B signaling is predominately important while PR-A remains important for the normal function of the uterus and ovary. As women’s menstrual cycle span an average of 36 years, they are constantly exposed to both ovarian steroid hormones E2 and P4 for prolonged periods of time [50]. Moreover, high serum P4 levels are observed during the luteal phase, when mammary terminal ductal cells are at their most proliferative state [51]. P4′s action on normal mammary gland is of importance for mammary cell proliferation and turnover, however prolonged exposure to P4 can lead to the dysregulation of various pathways that may lead to breast cancer [52]. Mammary gland proliferation is dominantly stimulated by P4 via both cell-intrinsic autocrine and paracrine mechanisms [53]. During mammary gland development PR-B specifically up regulates RANKL, a crucial P4 induced paracrine-signaling factor [54]. Furthermore, in a breast tissue microstructure ex vivo model, RANKL triggered cell proliferation and was required for P4-induced proliferation [55]. P4-induced cell proliferation can also be activated via paracrine signaling, which effects neighboring PR-negative mammary cells and largely relies on RANK and RANKL [55,56]. Interestingly P4 can stabilize RANKL mRNA expression and only PR+ cells elicit RANKL proteins [57,58]. P4-induced intrinsic proliferation by autocrine activation has been shown through the activation of the downstream target CCND1 (Cyclin D1). These data demonstrate that P4 is a key player in mammary gland proliferation and PR isoform expression could dictate site specific actions.

## 4. Non-Classical P4 Signaling

Steroid hormones are readily deemed to act on their nuclear receptors by classical signaling, however rapid progestin-activated signaling has been demonstrated by non-classical pathways [59]. In T47D cells, the progestin R5020 rapidly activated the epidermal growth factor receptor (EGFR), c-Src and MAPK-dependent phosphorylation of Ser345 on PRs which associates with transcription factors that can control *p21* and *EGFR* [59]. Furthermore, P4 can activate c-Src and enhance prolactin-mediated activation of signal transducer and activator of transcription (STAT) through MAPK pathways to promote cell proliferation [60] (Figure 1B).

Studies have demonstrated the ability for P4 signaling to occur through cell surface receptors in humans [61]. Here, progesterone does not have to diffuse through the plasma membrane but rather binds to receptors present on cellular membranes. It is now known that progestins have the ability to bind and activate several membrane-bound P4 receptors recognized as membrane progesterone receptors (mPRs), a class of 7 transmembrane domain proteins, structurally resembling G protein-coupled receptors (GPCRs); ligand-binding assays have demonstrated that these receptors have high affinity to P4 [62]. Five mPRs (mPRα, mPRβ, mPRɣ, mPRε and mPRδ) have been identified and they are known as progestin and adipoQ receptors (PAQRs) [63,64,65,66]. Upon P4 biding to its membrane receptors the signal transduction cascades activate MAPKs, ERK1/2 and intracellular Ca^2+^ influx [67,68,69]. (Figure 1C).

Specific tissue localization of mPR transcripts indicates their non-classical actions in mammals, as significant levels of mPR in human testes, ovary, placenta and breast have been described [61]. Further analysis revealed that mPRs have the ability to bind progestins with rapid dissociation and association rates, suggesting their role in mediating a rapid progesterone response [63]. Membrane-Associated Progesterone Receptors (MAPRs) that do not follow the same structural GPCR format have also been described, structurally they present as a single transmembrane domain type of protein known as progesterone receptor membrane component 1 and 2 (PGRMC1 and PGRMC2) [70]. Human PGRMC1 and PGRMC2 code for two membrane proteins each containing an N-terminal transmembrane segment and a C-terminal cytochrome b_5_ -like domain capable of binding penta-coordinated heme and are considered to be a member of the MAPR family of proteins [71,72,73]. PGRMC1 has been demonstrated to play an important role in cholesterol synthesis and has been shown to interact with cytochrome P450 (CYP) enzymes. PGRMC1 is capable of binding to SERBP1, together they act as rapid mediators for progestin actions, in various cells that lack the nuclear PR, demonstrating the effects of P4 on the cell through cell surface receptors [74]. Unlike PGRMC1, not much is known about PGRMC2. PGRMC2 has been shown to be primarily localized in the endoplasmic reticulum and nuclear envelope [73,75,76,77]. Similar to PGRMC1, PGRMC2 can also bind to CYPs, although studies have identified limited binding partners [78]. Recent studies have elucidated an important role for PGRMC2 in brown fat, and for intracellular heme transport to the nucleus [79]. In general, P4 actions are accomplished through the classical (genomic) and non-classical (nongenomic) signaling pathways.

## 5. P4 Classical Signaling in Breast Cancer

P4 is thought to exert its actions mainly through the binding of the classical nuclear PR. In general, PR-A and PR-B isoforms are expressed at similar levels in the normal breast epithelium while an imbalance of PR-A and PR-B ratio occurs early in breast cancer development and is commonly seen in premalignant breast lesions [80]. Studies have demonstrated distinct transcriptional activity between PR-A or PR-B isoforms that are dependent on P4 [46,81].

In the presence of P4, PR-B exhibits stronger transcriptional regulation compared to PR-A, however in the absence of P4, PR-A plays a more dominant role [82]. In breast cancers, expression of PR-A transcribes genes involved in cell proliferation and metastatic processes, of particular interest is *TNFRSF11A*, which encodes for the receptor of RANKL [83]. Disruption of RANKL has been associated with early stages of mammary tumor formation in a progestin responsive manner [84,85]. RANK, the receptor for RANKL, has also been demonstrated to be expressed in breast cancer cells and plays a fundamental role in the proliferation, differentiation and migration of these cells [86]. Furthermore, it has been shown that RANKL inhibitors can limit progestin-induced mammary carcinogenesis, while P4 mediated activation of RANKL enhances cell proliferation through the glioma-associated oncogene homolog 1 (GLI-1) via NF-kB/upstream stimulatory factor-1 (USF-1) [56,87]. Mechanistically, studies demonstrate that HIF-1 alpha can induce RANKL expression and promote the migration of breast cancer cells by the activation of PI3K/AKT signaling [88,89]. The RANKL/RANK signaling axis can also regulate the activation of cyclin D1 [90]. In ER+/PR+ cells, P4 promotes proliferation via a cyclin D1-dependent, cell-intrinsic mechanism and overexpression and amplification of Cyclin D1 correlates with poor prognosis in women diagnosed with ER+/PR+ breast cancers [91,92]. Furthermore, PR interaction with FOXO1 and CK2 coordinate a hormone dependent response to progestins allowing PR to interact with cyclin D1, enabling cell cycle regulation [85,93,94].

High levels of PR-A have been associated with a poor response or resistance to tamoxifen treatment [95,96]. In addition, the anti-progestin, mifepristone inhibits cell proliferation in studies with PR-A predominant tumors, but not in PR-B predominant tumors [97]. These findings suggest that the PR-A and PR-B ratio is an important indicator of response to different therapeutic options. Overexpression of PR-A in human breast cancer cells has also been shown to decrease cell adhesion and increase migration into bone marrow stroma [81,98,99]. Commonly observed features associated with neoplasia’s such as hyperplasia, disorganized basement membrane, and reduced cell-cell adhesion are also observed in PR-A overexpressing transgenic mouse models, which suggests that they are predisposed to develop mammary tumors [100].

### 5.1. Natural P4 vs. Synthetic Progestins

The role of P4 is controversial in terms of mammary cancers because synthetic progestins have been shown to be growth promoting, while natural P4 is thought to be growth inhibitory and it also plays an important role in normal reproductive physiology [101,102,103]. In the E3N-EPIC cohort an HRT study conducted in France, postmenopausal women who received micronized P4 with E2s showed no or minor increased risk of breast cancer [104]. However, others and our data show that P4 can promote breast cancers. We, demonstrated that in ovariectomized (OVX) August-Copenhagen Irish (ACI) rats, both E2 and P4 are required to induce mammary tumors [105]. While Shull et al. [106] demonstrated that in OVX ACI rats E2-alone treatment failed to induce mammary tumors. Furthermore, we also demonstrated that treatment with PR-antagonist, mifepristone significantly inhibits E2-induced mammary growth in vivo and P4 alone increased in vitro breast cancer cell proliferation [87]. These studies correlate with the WHI studies, which demonstrated the inability of E2 alone to increase the risk of breast cancer in women, and demonstrates the significance of progestogens [107].

### 5.2. Targeting PRs for Breast Cancer Treatment

Studies have demonstrated that PR can function and regulate selective target gene independently of ER, influencing mammary cancer cell proliferation and survival [83,108]. Therefore, targeting the PR in PR+ breast cancers may become a valid therapeutic option. In vivo and in vitro preclinical studies have demonstrated that PR function can be blocked by prototypical anti-progestogens known as selective progesterone receptor modulators (SPRMs), mifepristone or onapristone to control breast cancer progression [109,110,111]. Onapristone, is regarded as a possible first-line therapy in primary human breast cancers [112]. Moreover, anti-progestin activity is also observed by the more PR selective next-generation SPRM, telapristone acetate (TPA) which minimizes off target effects while inhibiting in vivo tumor growth and in vitro cell proliferation [113,114,115,116]. Therefore, targeting PRs with SPRMs could become an option for patients presenting with PR+ tumors.

## 6. Non-Classical P4 Signaling in Breast Cancer

Non-classical signaling can also contribute to breast cancer pathology; studies have demonstrated an up regulation of mPRα and PGRMC1 in breast cancer cell lines [117]. The overexpression of theses receptors in both ER+/PR+ and TNBC cells and tissues indicates that P4 could affect both luminal and basal-like breast cancers [117,118]. Both mPRα and PGRMC1 are observed in primary breast tumor tissues [119,120]. P4 signaling has been observed in TNBCs, through mPRα and mPRβ [121]. The effects of P4 on mPRs are controversial. Studies by Zhou et al. [122] describe the ability of P4 to suppress the growth and metastasis of MDA-MB-468 TNBC cells to the brain through mPRα. P4 has also been described to inhibit cell proliferation of MDA-MB-231 cells and reverse the mesenchymal phenotype of these cells to epithelial-like phenotypes through mPRα [123]. P4 has been reported to inhibit TNBC cell proliferation and migration through mPRα and Src/focal adhesion kinase (FAK) signaling [123]. However, P4 treatment to PR-negative SKBR3 and MDA-MB-468 cells activates MAPK, p42 and AKT signaling pathways while inhibiting apoptosis through multiple mPRs [118]. Further, positive correlation between mPRα, p-AKT and EGFR levels have been described in breast cancer cells [120,124]. Larger tumor size and lymph node metastasis were found to correlate with overexpression of PGRMC1 and patients observed with mPRα and PGRMC1 expressing tumors have poor disease-free and overall survival [119,120]. Furthermore, progestogens may be capable of transforming normal breast cells into cancerous cells as evidence from the P4 metabolites, 5α-dihydroprogesterone (5αP), 3α-dihydroprogesterone (3αHP) and MPA which were capable of activating ERK, c-Jun N-terminal kinase (JNK) and AKT signaling in non-malignant breast epithelial MCF12A cells [125]. Treatment of the metabolites 5αP, 3αHP and MPA also promoted cell proliferation of MCF10A breast epithelial cells through multiple mPRs and PGRMC1 [125]. The confirmation of mutations that could occur following P4 treatment of normal breast cells remains to be explored. Overall, P4s actions via non-classical signaling is mediated through multiple membrane receptors that may influence the growth and progression of breast cancers.

### Role of Progesterone Receptor Membrane Component 1 (PGRMC1) in Breast Cancer

Specific cancer characteristics such as, EMT, chemotherapy resistance, motility, anchorage-dependent growth, vascular endothelial growth factor (VEGF)-induction and metastasis are all influenced by PGRMC1 [126]. Moreover, PGRMC1-KD enhanced sensitivity to the anti-cancer drug, doxorubicin [71]. Cell impermeable bovine serum albumin-fluorescein isothiocynate (BSA-FITC) conjugated P4 was used to demonstrate increased cell proliferation of MCF7 cells via non-classical signaling [127]. Following PGRMC1 overexpression in MCF7 and T47D cells, norethisterone (NET) treatment caused PGRMC1 phosphorylation which led to increased cell proliferation [128]. In PGRMC1 transfected T47D and MCF-xenotransplants, the combination of E2 and NET resulted in increased PGRMC1 expression [129]. Interestingly, PGRMC1 has been shown to bind and stabilize EGFR [17]. This is significant because EGFR overexpression has been observed in multiple tumor tissues and is associated with overall poor prognosis [130,131,132,133]. Activation of EGFR has been associated with increased proliferation, metastasis and angiogenesis [134]. Interaction between PGRMC1 and EGFR has been shown to promote tumor growth [17]. AG-205 (PGRMC1 inhibitor) treatment decreased both total and phosphorylated EGFR leading to a decrease in breast cancer cell proliferation [17,135,136,137]. PGRMC1 has been shown to be increased in breast cancer cell lines and breast tumor tissue, however the mechanism behind its role in breast cancers remains elusive.

## 7. Conclusions

**Figure 2:**
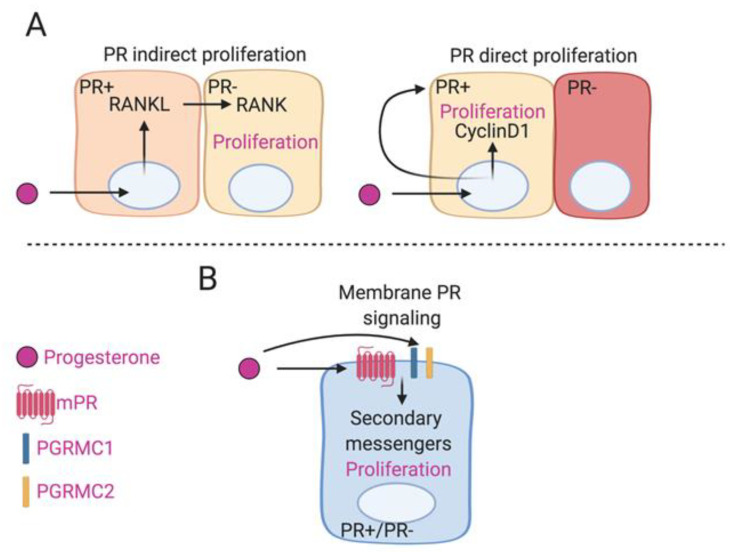
Progesterone-induced mammary proliferation by classical and non-signaling. (**A**) The majority of the mammary gland consists of PR negative luminal epithelial cells. However, progesterone is capable of inducing sustained proliferation of PR-negative luminal epithelial cells by indirect paracrine signaling whereby PR-positive cells upregulate RANKL and act on RANK in PR-negative adjacent cells. PR-positive cells can also exhibit progesterone induced proliferation by self-intrinsic activation of cyclinD1 via autocrine signaling. (**B**) Progesterone-induced mammary proliferation by non-classical signaling. Progesterone can act on a variety of membrane progesterone receptors such as mPRs, PGRMC1 and PGRMC2 that can activate secondary messengers and promote the proliferation of either PR-positive or PR-negative mammary cells. Figure 2 was created using Biorender.

Progesterone has the ability to bind to both nuclear and membrane receptors activating classical (Figure 2A) and non-classical (Figure 2B) downstream signaling pathways to promote cell proliferation and tumor growth. Historically, the PR has merely been considered a molecular marker for functional ER activity. However, most patients who present ER+ also present PR+, a combination of anti-E2 plus anti-progestogen treatment may allow for better clinical outcomes, especially for patients presenting with ER+ anti-E2 resistant tumors. PGRMC1 has the potential to be a viable biomarker for clinical diagnosis of breast cancers. PGRMC1 has been associated with strong membrane expression in human breast tumor tissue [138]. Immunostaining can be utilized to differentiate between nuclear and membrane staining of PR and PGRMC1 respectively and assigning intensity scores could better distinguish PR and PGRMC1 expression in breast cancer tumors [139]. Furthermore, artificial intelligence along with machine learning software such as Nuquantus, that can be trained for accurate and rapid classification of cell subtype nuclei after studying and identifying patterns of tissue architecture could be trained to specifically identify and distinguish between nuclear and membrane staining [140]. Immunohistochemistry, immunofluorescence along with machine learning and staining intensity software could be used to identify PGRMC1 in human breast cancer tissues. In conclusion, progesterone-associated membrane receptors are widely expressed in breast cancer cell lines and tumor tissue and can facilitate the growth of breast cancer cells in vitro and tumor formation in vivo and should be considered viable therapeutic targets and biomarkers for breast cancers.

## Abbreviations

E2	Estrogen
ER	Estrogen receptor
P4	Progesterone
PR	Progesterone receptor
TNBC	Triple negative breast cancer
WHI	Women’s health initiative
HRT	Hormone replacement therapy
HER2	Human epidermal growth factor receptor 2
MPA	Medroxyprogesterone acetate
RANKL	Receptor activator of nuclear factor kappa-B ligand
RANK	Receptor activator of nuclear factor kappa-B
CCND1	Cyclin D1
HRE	Hormone response element
PRE	Progesterone response element
EGFR	Epidermal growth factor receptor
MAPK	Mitogen activated protein kinase
STAT	Signal transducer and activator of transcription
mPR	Membrane progesterone receptor
GPCR	G protein coupled receptor
PAQR	Progestin and adipoQ receptor
MAPR	Membrane associated progesterone receptor
PGRMC1	Progesterone receptor membrane component 1
PGRMC2	Progesterone receptor membrane component 2
GLI-1	Glioma associated oncogene homolog 1
NF-kB	Nuclear factor kappa B
OVX	Ovariectomized
ACI	August copenhagen irish
SPRM	Selective progesterone receptor modulator
TPA	Telapristone acetate
NET	Norethisterone
CK2	Casein kinase 2
5αP	5α-dihydroprogesterone
3αHP	3α-dihydroprogesterone
